# Identification of Alp1U and Lom6 as epoxy hydrolases and implications for kinamycin and lomaiviticin biosynthesis

**DOI:** 10.1038/ncomms8674

**Published:** 2015-07-02

**Authors:** Bin Wang, Fang Guo, Jinwei Ren, Guomin Ai, Bertrand Aigle, Keqiang Fan, Keqian Yang

**Affiliations:** 1 State Key Laboratory of Microbial Resources, Institute of Microbiology, Chinese Academy of Sciences, Beijing 100101, China; 2 College of Life Sciences, University of Chinese Academy of Sciences, Beijing 100049, China; 3 State Key Laboratory of Mycology, Institute of Microbiology, Chinese Academy of Sciences, Beijing 100101, China; 4 Université de Lorraine, Dynamique des Génomes et Adaptation Microbienne, UMR 1128, Vandœuvre-lès-Nancy F-54506, France; 5 INRA, Dynamique des Génomes et Adaptation Microbienne, UMR 1128, Vandœuvre-lès-Nancy F-54506, France

## Abstract

The naturally occurring diazobenzofluorenes, kinamycins, fluostatins and lomaiviticins, possess highly oxygenated A-rings, via which the last forms a dimeric pharmacophore. However, neither the A-ring transformation nor the dimerization mechanisms have been explored thus far. Here we propose a unified biosynthetic logic for the three types of antibiotics and verify one key reaction via detailed genetic and enzymatic experiments. Alp1U and Lom6 from the kinamycin and lomaiviticin biosynthesis, respectively, are shown to catalyse epoxy hydrolysis on a substrate that is obtained by chemical deacetylation of a kinamycin-pathway-derived intermediate. Thus, our study provides the first evidence for the existence of an epoxy intermediate in lomaiviticin biosynthesis. Furthermore, our results suggest that the dimerization in the lomaiviticin biosynthesis proceeds after dehydration of a product generated by Lom6.

## Abstract (web summary)

Kinamycins, fluostatins and lomaiviticins are naturally occurring antibiotics that possess unique diazofluorene structures and exhibit potent anti-tumour activity. Here the authors identify the epoxy hydrolases in the biosynthetic pathways of kinamycins and lomaiviticins, suggesting the existence of epoxy-intermediates in their biosynthesis.

## Graphical abstract

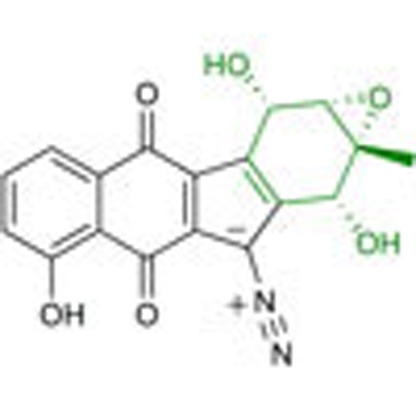

Antibiotics of the kinamycin class, including the kinamycins^1^, fluostatins^2^,^3^ and lomaiviticins^4^,^5^, possess three unique functionalities generated in one assembly line, that is, the benzofluorene core, the diazo group and the highly oxygenated A-ring; all three functionalities were reported to be potentially connected to their novel antibiotic and antitumour activities^6^,^7^,^8^,^9^,^10^. Recently, lomaiviticins attracted renewed interest due to their dimeric structure joined by a C–C bond between kinamycin-like monomers. Also because among lomaiviticins, lomaiviticin A was demonstrated to exhibit potent cytotoxicity at nM–pM concentrations^11^. The biosynthetic machineries forming the benzofluorene core and diazo group were proposed to be shared by all three pathways, and formation of the benzofluorene in the *alp* cluster (for the kinamycin biosynthesis) has recently been elucidated by our group^12^. The highly oxygenated A-rings, however, reflect their evolutionary divergence, that is, a four-oxygen-substituted (tetra-hydroxyl) A-ring in kinamycins and three-oxygen-substituted A-rings in fluostatins and lomaiviticins, which undergo further modifications such as acylation, glycosylation or dimerization.

From careful inspection of the diverse structures of the kinamycins and fluostatins, combined with our present results, we propose a unified biosynthetic logic for construction of the oxygen-substituted A-rings, and propose candidate enzymes (Fig. 1). To verify our hypothesis in the kinamycin and lomaiviticin pathways, we focused on one key enzyme, the epoxy hydrolase, whose product not only could confirm the existence of an epoxy intermediate and an epoxidase accordingly but also shed light on the dimerization precursor. We extended the previously proposed incomplete *alp* cluster^13^,^14^ in *Streptomyces ambofaciens* to the far side of the genome to include region 1 and proved that an epoxy hydrolase, Alp1U, is responsible for the epoxy opening on epoxykinamycin (**1**) via detailed genetic and enzymatic analyses. Lom6, as the predicted counterpart of Alp1U in the lomaiviticin biosynthesis, was subsequently analysed and proved to work on **1**, suggesting that a monomeric epoxy intermediate also exists in the lomaiviticin biosynthesis and undergoes a similar epoxy opening process as predicted. The results presented here allow us to revise previous predictions for the lomaiviticin A-ring transformation, and facilitate future investigation of the dimerization reaction.

## Results

### Extension of the *alp* gene cluster

We noted that the previously predicted *alp* cluster^14^ is incomplete, not only for lacking the genes of the diazo assembly machinery but also for genes involved in the A-ring transformation. As predicted by Gould^15^,^16^, the kinamycin A-ring transformation from prekinamycin to ketoanhydrokinamycin proceeds similarly to the biosynthesis of antibiotic LLC10037a (Supplementary Fig. 1). Thus, at least two oxygenases or an additional reductase is required. We further hypothesized that an epoxy hydrolase should be necessary to generate the tetra-hydroxyl kinamycin F (**2**). However, all oxygenases in the previously proposed *alp* cluster have already been assigned functions. Consequently, we extended the *alp* cluster to include region 1 (SAMT0157-0134) via comparative bioinformatic analysis (Supplementary Fig. 2 and Supplementary Table 1). Region 1 was further divided into five subregions, among which the subregion from *alp1A* to *alp1H* is speculated to be involved in the detoxification reactions (activating the mycothiol biosynthetic pathway to *S*-conjugate the toxic intermediates)^17^ and DNA damage–repair pathways; the subregion from *alp1I* to *alp1N* is highly conserved among the *alp*, *flu* and *lom* clusters (Supplementary Fig. 2), thus it is proposed to be involved in the diazo assembly; the subregion from *alp1O* to *alp1R* is mainly composed of transporters; and the subregion from *alp1S* to *alp1V* is a seemingly independent subset of four genes whose annotations suggest they encode enzymes for kinamycin A-ring transformation; and the subregion including *alp1W* and *alp1X* is predicted to be involved in diazo formation as well.

Phylogenetic analysis of the oxygenases in the *alp*, *flu* and *lom* gene clusters demonstrates that the enzymes predicted to be involved in the A-ring transformation (Alp1S and Alp1V, Flu21 and Flu29, and Lom16 and Lom17, respectively) with the exception of Flu29 are clustered in a branch distinct from those well-characterized oxygenases involved in biosynthesis of angucycline antibiotics, suggesting that these oxygenases (represented by Alp1S and Alp1V) catalyse similar A-ring transformation reactions, that is, a C4-hydroxylation and a C2, C3-epoxidation, and that Flu29 is probably the epoxidase working on an isomerized fluostatin intermediate (Supplementary Fig. 3). In addition, a substrate-based clustering is also observed by the neighbour joining of enzymes from the same gene clusters (Alp1S with Alp1V, and Lom16 with Lom17). Stand-alone BLAST was then performed to re-analyze all proteins coded by the three gene clusters to identify the counterparts of Alp1T and Alp1U. Combined with visual inspection of the conserved domain(s), Flu20 and Flu24, and Lom6 and Lom19, were putatively assigned.

The next step is predicted to be a C1-, C4-keto reduction catalysed by short-chain dehydrogenase/reductase (SDR) family proteins. Sequence alignment shows that the three NDP-dependent epimerases, Alp1T, Flu20 and Lom19, share a common SDR domain but display rather low identities (∼30%, enzymes of the SDR superfamily typically show low sequence identity in the 15–30% range^18^). Lom19 was recently predicted to be the dimerization enzyme, based on similarity to ActVA-orf4 (refs 4, 5), but evidence for this is still lacking.

The structure diversity of A-rings of the kinamycins and fluostatins led us to hypothesize the existence of an epoxy A-ring intermediate in the lomaiviticin biosynthetic pathway, and accordingly an epoxidase and an epoxy hydrolase. A conserved domain search demonstrates that Alp1U, Flu24 and Lom6 all belong to the α/β-hydrolase family, but the pairwise sequence alignment shows that Flu24 and Lom6 display high similarity (62%) but differ remarkably from Alp1U (28 and 25% similarity, respectively), which is consistent with our prediction that an epoxy hydrolysis reaction on the A-ring of fluostatins and lomaiviticins should be followed by a dehydration reaction, resulting in the mono-hydroxyl group at C2 and C3, respectively, rather than the di-hydroxyl found on kinamycins. Accordingly, the C4-keto in fluostatins and C1-keto in lomaiviticins could be explained by keto–enol tautomerization following epoxy hydrolysis and dehydration. These reactions would generate the dimerization precursor in the lomaiviticin biosynthesis, which would then be dimerized by an as yet unidentified phenol-coupling oxidase^19^,^20^.

### Functional analysis of Alp1U

Since the *alp* cluster (including region 1) is located in the chromosomal terminal inverted repeats in *S. ambofaciens*^13^,^14^, both copies of *alp1U* were first in-frame deleted to investigate the function of the epoxy hydrolases (Supplementary Fig. 4). The mutant, ΔΔ*alp1U*, was unable to produce kinamycin D (**3**), but accumulated a yellow compound (**4**) instead (Fig. 2). Liquid chromatography–mass spectrometry (LC–MS) analysis suggested it to be epoxykinamycin FL-120B′ (*m/z* 393 [M-H]^−^), which is a known compound previously isolated from *Streptomyces chattanoogensis*^21^ and also detectable in trace quantities in our starting strain^13^. After large-scale fermentation and purification, ^1^H, ^13^C NMR data of **4** were collected and compared with the previously reported data^21^. Minor variations in chemical shift were detected, thus we performed NMR again to further collect COSY, QC, HMBC and NOESY data and *de novo* characterized **4**. In the structure of **4**, C2, C3-epoxy A-ring and *O*-acetylated C4-hydroxyl group were observed. The latter was unexpected according to current predictions^1^,^4^,^5^. The production of **3** was restored by introduction of one copy of *alp1U* to ΔΔ*alp1U* (Fig. 2), which confirmed that Alp1U functions as epoxy hydrolase *in vivo*. With **4** in hand as a potential substrate for Alp1U, N-terminal His_6_-tagged Alp1U was expressed in *Escherichia coli* and purified to near homogeneity (Supplementary Fig. 5). When **4** was incubated with Alp1U, a more hydrophilic compound (**5**) was produced (Fig. 3). Subsequent LC–MS analysis of **5** suggested it to be the predicted intermediate, kinamycin E (Supplementary Fig. 6)^1^. We then performed the reaction on a larger scale to obtain sufficient amount of **5** for NMR measurements. Although some difficulties such as solvent selection, compound conversion (Supplementary Fig. 7) and variations in chemical shift were encountered, adequate NMR data could be collected for *de novo* assignment of **5**. NOESY further confirmed the chirality of C2 is the same as kinamycin E^22^.

### Enzymatic analysis of Lom6

**Figure 1:**
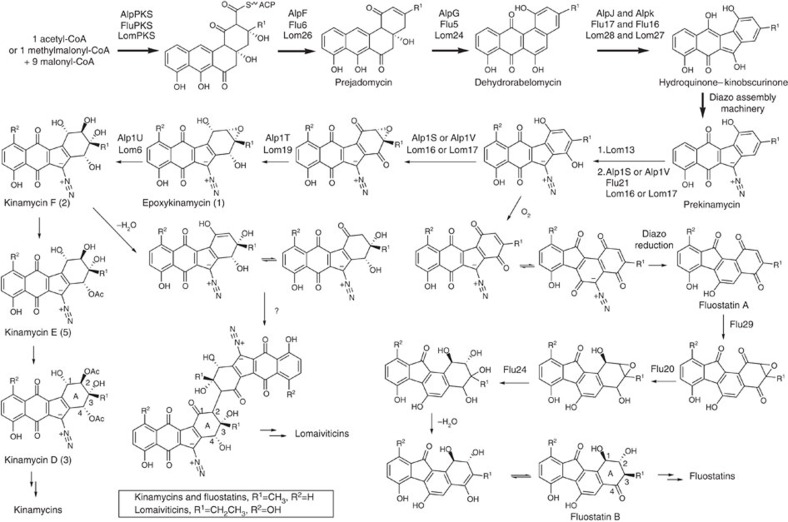
The proposed biosynthetic pathway for kinamycin class antibiotics.

**Figure 2:**
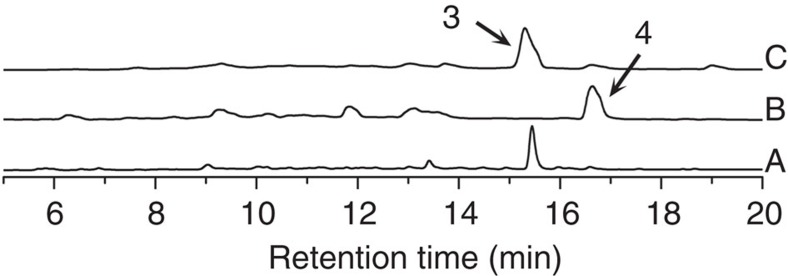
HPLC profiles of crude extracts from mutants studied in this work. Trace A, the starting strain producing kinamycin D (**3**); trace B, ΔΔ*alp1U* producing epoxykinamycin FL-120B′ (**4**); trace C, ΔΔ*alp1U*::*alp1U* restoring the production of **3**.

**Figure 3:**
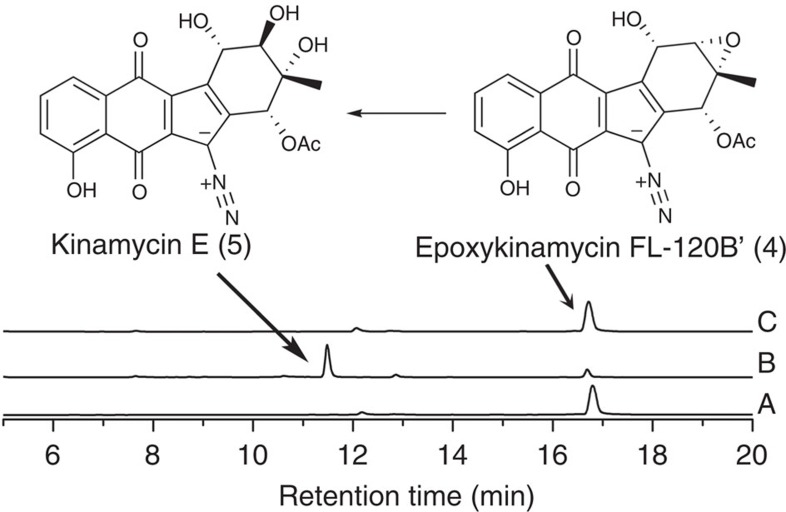
HPLC analysis of enzymatic reactions. Trace A, standard epoxykinamycin FL-120B′ (**4**); trace B, **4** with Alp1U, producing kinamycin E (**5**); trace C, **4** with Lom6.

**Figure 4:**
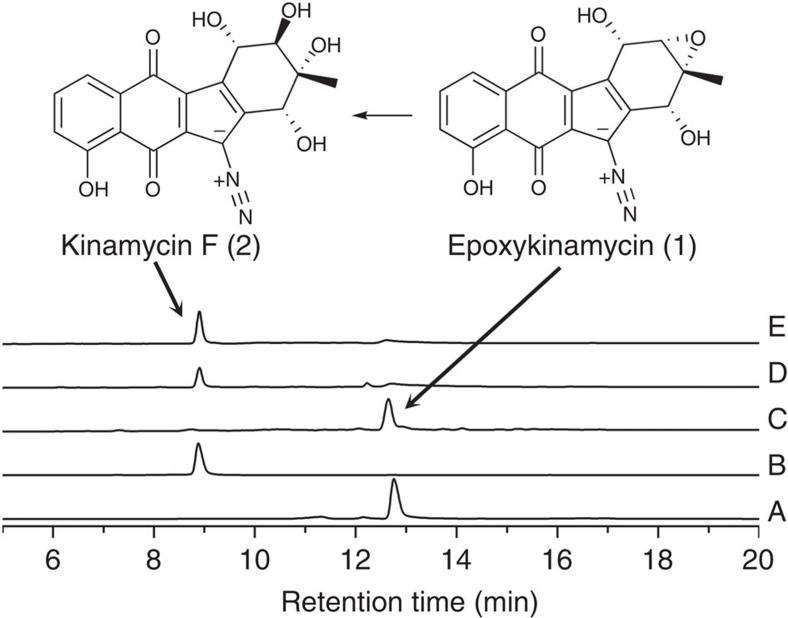
HPLC traces of enzymatic reactions. Trace A, epoxykinamycin FL-120B′ (**4**) treated with 0.1 N LiOH, forming epoxykinamycin (**1**); trace B, kinamycin E (**5**) treated with 0.1 N LiOH forming kinamycin F (**2**); trace C, standard **1**; trace D, **1** with Lom6 in 50 min producing **2**; trace E, **1** with Alp1U in 10 min producing **2**.

Next, to confirm the hypothesis of an epoxy intermediate in the lomaiviticin biosynthesis pathway, we synthesized *lom6* and expressed the protein in *E. coli* (Supplementary Fig. 8). The kinamycin-pathway-derived intermediate **4** was used as substrate to test the activity of Lom6. The same reaction conditions used for Alp1U were applied to Lom6, but high-performance liquid chromatography (HPLC) analysis did not provide any evidence for the conversion of **4** by Lom6, even following longer incubation (Fig. 3). This suggests there are structural differences between the structures of **4** and the genuine Lom6 substrate. The C4-*O*-acetyl group is the most conspicuous extra moiety since no acetyl groups was reported on the lomaiviticin antibiotics. Therefore, **1** and **2** were prepared by alkaline hydrolysis of **4** and **5** (Fig. 4), and confirmed by High Resolution Mass Spectrometry (HRMS). **1** is a new compound predicted in Fig. 1 we found to be unstable and degrading within hours. In further tests, **1** could be converted to **2** by Lom6 (Fig. 4), which was confirmed by LC–MS (Supplementary Fig. 9). The same reaction was immediately set for Alp1U, and HPLC analysis indicated that Alp1U converted **1** to **2** (Fig. 4 and Supplementary Fig. 10). These results demonstrate that Lom6 can also act upon **1** as an epoxy hydrolase, but exhibits lower efficiency on the kinamycin-pathway-derived substrate **1**. Incompatibility between **1** and Lom6 might explain those observations, but it is also possible that epoxy hydrolysis catalysed by Lom6 is coupled with dehydration in the lomaiviticin biosynthesis.

## Discussion

The above results clearly demonstrate that Alp1U and Lom6 are epoxy hydrolases. However, it remains surprising that the C4-*O*-acetylation—which is supposed to occur after epoxy hydrolysis—does not block Alp1U-catalysed epoxy hydrolysis. As previously reported, both **2** and **5** are confirmed to be intermediates in kinamycin biosynthesis^1^, thus our observation that Alp1U converts both **1** to **2** and **4** to **5** implies that **1** and **4** are intermediates and that the order of the last few steps in the biosynthesis of kinamycin—proximal to the epoxy hydrolysis and *O*-acetylation—are not strictly fixed.

In conclusion, facilitated by comparative bioinformatic analysis we are able to deduce a unified scheme for the A-ring transformation of antibiotics of the kinamycin class (Fig. 1), in which for the first time epoxyquinol intermediates in the kinamycin and lomaiviticin biosynthesis are proposed. We have also demonstrated that Alp1U and Lom6 are epoxy hydrolases that can catalyse the hydrolysis of a kinamycin-pathway-derived epoxy intermediate, suggesting the existence of a similar epoxy intermediate—hence also an epoxidase—in the lomaiviticin pathway. Further work will aim to identify the phenol-coupling oxidase responsible for the dimerization reaction in the lomaiviticin biosynthetic pathway.

## Methods

### General

All cultures of *E. coli* were grown in Luria–Bertani medium supplemented with appropriate antibiotics at 37 °C. *E. coli* JM109 was used as host for subcloning, ET12567/pUZ8002 as donor for intergeneric conjugation and BL21(DE3) for protein expression. *S. ambofaciens* ΔΔ*alpW* as the starting strain and their derivatives were maintained on MYM solid medium for sporulation, on MS solid medium for conjugation, in R2 liquid medium for metabolite production and in YEME liquid medium for preparing genomic DNA at 28 °C (ref. 23).

HPLC was carried out on an analytical column Agilent ZORBAX SB-C18 (4.6 × 250 mm, 5 μm) connected to a Shimadzu LC-20AT system equipped with a diode array detector monitoring absorbance at 223 and 276 nm. Elution was performed with a linear gradient from 25 to 100% acetonitrile in water containing 0.1% trifluoroacetic acid (TFA) for 20 min at a flow rate of 1.0 ml min^−1^.

LC–MS was performed on an Agilent 1260/6460 Triple Quadrupole LC/MS system, using an Agilent ZORBAX SB-aq C18 column (2.1 × 100 mm, 3.5 μm). High-resolution mass spectrometry measurements were carried out on an Agilent 1200HPLC/6520 Q-TOF-MS mass spectrometer (Agilent Technologies Inc.) within 0.4 p.p.m. errors between theoretical and measured values. All NMR experiments were acquired on a BrukerAvance 500 MHz (*B*_*0*_11.74 T) spectrometer in (CD_3_)_2_SO equipped with a 5 mm Prodigy probe, using TMS as internal standard.

Bioinformatic analysis was performed, using MEG4.0 Neighbour-Joining method for phylogenetic tree construction, using Stand-alone BLAST (download from http://www.ncbi.nlm.nih.gov/guide/sequence-analysis/) for alignment of the three gene clusters (*alp*, *flu and lom*), and using the net-based National Center for Biotechnology Information Conserved Domain Search Service for deduction of protein functions.

### Construction of *S. ambofaciens* ΔΔ*alp1U*

For in-frame deletion of *alp1U*, two homologous fragments were amplified by PCR from *S. ambofaciens* ΔΔ*alpW* genomic DNA with primers 1U-upF, 1U-upR, 1U-dnF and 1U-dnR (sequences and restriction sites in Supplementary Table 4). The PCR products were purified and digested by the corresponding restriction enzymes before ligated to the pre-digested thermal-sensitive plasmid pKC1139, yielding the gene knockout plasmid pKC1139-Dalp1U. The integrity of the insert was confirmed by sequencing at Invitrogen. Then the resulting plasmid was introduced into the starting strain by conjugation from *E. coli* ET12567/pUZ8002. The transformants were cultured at 28 °C for two successive generations to enable double-crossover events to occur and then transferred to 37 °C for plasmid loss. Gene knockout was analysed by PCR using the flanking primers CK-1U-F and CK-1U-R (sequences in Supplementary Table 4).

### Complementation of *S. ambofaciens* ΔΔ*alp1U*

For complementation analysis, the integrative plasmid pSET616, a pSET152 derivative, containing the promoter SF14 and *neo* reporter gene was used. The *alp1U* coding sequence plus its ribosome-binding site was PCR amplified using primers 1U-F and 1U-R (sequences and restriction sites in Supplementary Table 4), and then ligated into the pre-digested pSET616 to create pSET-alp1U. Proper construction of pSET-alp1U was confirmed by DNA sequencing at Invitrogen. The complementation plasmid was then conjugated into ΔΔ*alp1U* to generate ΔΔ*alp1U*::*alp1U*. The transformants were cultured at 28 °C for several successive generations to isolate single colony.

### Extraction and preparation of 4

For preparation of **4**, a 4-l fermentation broth of the mutant ΔΔ*alp1U* was extracted by ethyl acetate after pH was adjusted to 3–4. The ethyl acetate was removed by vacuum evaporation, and the extract was dissolved in methanol, and then applied to the Sephadex LH-20 chromatography using methanol as eluting solvent. The fractions containing **4** were collected and pooled, and further purified by reverse-phase semi-preparative HPLC (YMC-Pack Pro C18, 250 × 10 mm, 5 μm; 4 ml min^−1^, ultraviolet detection at 223 and 276 nm) with an isocratic flow of 50% acetonitrile in water containing 0.1% TFA. At last, 3.0 mg of **4** was dissolved in (CD_3_)_2_SO for NMR measurements (Supplementary Figs 11–16 and Supplementary Table 2).

Epoxykinamycin FL-120B′ (**4**): yellow powder; ^1^H NMR ((CD_3_)_2_SO, 500 MHz) *δ* 12.07 (1H, s, 7-OH), 7.72 (1H, st, *J*=7.5 Hz, H-9), 7.63 (1H, d, *J*=7.5 Hz, H-10), 7.31 (1H, d, *J*=7.5 Hz, H-8), 6.34 (1H, s, H-4), 5.69 (1H, s, 3-OH), 5.06 (1H, s, H-1), 3.52 (1H, s, H-2), 2.26 (3H, s, H_3_-14), 1.40 (3H, s, H_3_-12); ^13^C NMR ((CD_3_)_2_SO, 125 MHz) *δ* 182.8 (qC, C-6), 180.2 (qC, C-11), 171.0 (qC, C-13), 161.6 (qC, C-7), 137.0 (CH, C-9,), 134.1 (qC, C-10a), 131.8 (qC, C-11a), 130.2 (qC, C-11b), 129.5 (qC, C-4a), 128.6 (qC, C-5a), 124.9 (CH, C-8), 120.3 (CH, C-10), 115.6 (qC,C-6a), 77.4 (CH, C-5), 68.8 (qC, C-4), 63.4 (CH, C-1), 61.6 (CH, C-2), 58.4 (qC, C-4a), 20.4 (CH_3_, H_3_-14), 19.39 (CH_3_, H_3_-12); HMBC data ((CD_3_)_2_SO, 500 MHz) H-1 →C-4a; 1-OH→C-1, C-2; H-2 →C-1, C-3, C-11b, C-12; H-4→C-1, C-3, C-11b, C-12; 7-OH→C-6a, 7, 8; H-8→C-6a, 7, 10; H-9→C-7, 10a; H-10→C-6a, 8; H_3_-12→C-2, 3, 4; H_3_-14→C-13; NOESY data ((CD_3_)_2_SO, 500 MHz): H-1↔H-2, H-4, H_3_-12; H-2↔H_3_-12; H-4↔H_3_-12; 7-OH↔H-8; H-8↔H-9; H-9↔H-10. ESIMS (*m/z*): [M-H]^−^ 393.

### Protein expression and purification in *E. coli* BL21(DE3)

The *alp1U* coding sequence was amplified by PCR from *S. ambofaciens* ΔΔ*alpW* genomic DNA using primers Ealp1U-F and Ealp1U-R (sequences and restriction sites in Supplementary Table 4). The *lom6* coding sequence was synthesized after codon optimization by Biomed. After digestion with the corresponding restriction enzymes, the DNA fragments were subcloned into pET28a and expressed with an N-terminal His_6_ fusion tag. The sequences were confirmed by sequencing in Invitrogen. Then the constructs were transformed into *E. coli* BL21(DE3). Protein expression was induced with 0.1 mM isopropyl-β-D-thiogalactopyranoside when the cultures were grown at 37 °C until the OD_600_ 0.4–0.6. And allow the cultures to grow at 20 °C for an additional 12 h. For protein purification, cells were harvested by centrifugation (8,000*g*) at 4 °C, and resuspended in 1 × binding buffer (500 mM NaCl, 20 mM Tris-HCl, 5 mM imidazole, pH 7.0 for Alp1U and pH 7.9 for Lom6). Then cells were disrupted by sonication (total time 10 min, 2 s cooling, 2 s burst at 250 W). Following centrifugation, the supernatant was loaded onto the prewashed Ni-NTA column. The crude proteins were washed firstly with 1 × wash buffer (500 mM NaCl, 20 mM Tris-HCl, 60 mM imidazole, pH as mentioned above) and then eluted with 0.5 × elute buffer (500 mM NaCl, 20 mM Tris-HCl, 500 mM imidazole, pH as mentioned above). The purified proteins were concentrated by centrifugation (4,000*g*, 30 min) in 10 kDa ultrafiltration tubes (Centriplus YM series, Millipore). Protein concentrations were determined by Bradford method^24^ and the purities were assessed by 15% SDS–PAGE.

### *In vitro* enzyme reactions

All reactions set for Alp1U was performed in 50 mM Tris-HCl buffer, pH 7.0, and for Lom6 in buffer pH 8.0. The enzyme concentrations were 25 μM for Alp1U and ∼20 μM for Lom6. The Alp1U reaction was incubated at 30 °C for 10 min, and Lom6 reactions varied from 10 to 50 min. Substrates, epoxykinamycin FL-120B′ (**4**) and epoxykinamycin (**1**), were not quantified but basically of the same quantity in all parallel reactions.

For preparation of kinamycin E (**5**), the reaction volume was amplified to 200 μl, incubation time increased to 30 min. Product was extracted by ethyl acetate before purified by semi-preparative column on HPLC. The HPLC gradient was changed to 25–85% acetonitrile in water containing 0.1% TFA in 16 min. Finally, 1.0 mg of **5** was dissolved in CD_3_Cl or ∼2.0 mg in (CD_3_)_2_SO for NMR experiments (Supplementary Figs 17–20 and Supplementary Table 3).

Kinamycin E (**5**): yellow powder; ^1^H NMR ((CD_3_)_2_SO, 500 MHz) *δ* 12.12 (1H, s, 7-OH), 7.70 (1H, t, *J*=8.5 Hz, H-9), 7.61 (1H, d, *J*=8.5 Hz, H-10), 7.29 (1H, d, *J*=8.5 Hz, H-8), 5.76 (1H, s, H-4), 5.26 (1H, d, *J*=6.0 Hz, 2-OH), 5.18 (1H, d, *J*=3.5 Hz, 1-OH), 4.50 (1H, dd, *J*=3.5, 6.0 Hz, H-1) 3.87 (1H, t, *J*=6.0 Hz, H-2), 2.10 (3H, s, H_3_-14), 1.09 (3H, s, H_3_-12); ^13^C NMR ((CD_3_)_2_SO, 125 MHz) *δ* 183.2 (qC, C-6), 180.2 (qC, C-11), 171.3 (qC, C-13), 161.6 (qC, C-7), 136.9 (CH, C-9,), 134.3 (qC, C-10a), 132.4 (qC, C-4a), 132.0 (qC, C-11b), 131.4 (qC, C-11a), 128.9 (qC, C-5a), 124.6 (CH, C-8), 119.9 (CH, C-10), 115.8 (qC,C-6a), 74.8 (CH, C-2), 73.2 (qC, C-3), 70.8 (CH, H-4), 69.3 (CH, H-1), 21.1 (CH_3_, H_3_-14), 20.6 (CH_3_, H_3_-12); HMBC data ((CD_3_)_2_SO, 500 MHz) H-1→C-3, 4a; H-2→C-1, 3, 11b, 12; H-4→C-2, 5, 11b, 12; 7-OH→C-6a, 7, 8; H-8→C-6a, 7, 10; H-9→C-7, 10a; H-10→C-6a, 8; H_3_-12→C-2, 3, 4; H_3_-14→C-13; NOESY data ((CD_3_)_2_SO, 500 MHz): H-1↔H_3_-12; H_3_-12↔H-4; 7-OH↔H-8; H-8↔H-9; H-9↔H-10. ESIMS (*m/z*): [M-H]^−^ 411.

### Preparation of epoxykinamycin (1) and kinamycin F (2)

An assay mixture (50 μl) composed of substrate **4** or **5** dissolved in MeOH and LiOH (0.1 M) was incubated at room temperature for ∼5 min. Then the crude product **1** or **2** was desalted using C18 column and analysed by HPLC. High Resolution Mass Spectrometry (HRMS) (*m/z*): for **1**, [M-H]^−^ calcd. for C_18_H_11_N_2_O_6_, 351.0695, found 351.0692; for **2**, [M-H]^−^ calcd. for C_18_H_13_N_2_O_7_, 369.0801, found 369.0799.

## Additional information

**How to cite this article:** Wang, B. *et al.* Identification of Alp1U and Lom6 as epoxy hydrolases and implications for kinamycin and lomaiviticin biosynthesis. *Nat. Commun.* 6:7674 doi: 10.1038/ncomms8674 (2015).

## Supplementary Material

Supplementary Information Supplementary Figures 1-20 and Supplementary Tables 1-4

## References

[b1] Gould S. J. Biosynthesis of the kinamycins. Chem. Rev. 97, 2499–2510 (1997). 11851467 10.1021/cr9600215

[b2] Zhang W. *et al.* Fluostatins I-K from the South China Sea-derived Micromonospora rosaria SCSIO N160. J. Nat. Prod. 75, 1937–1943 (2012). 23136829 10.1021/np300505y

[b3] Feng Z., Kim J. H. & Brady S. F. Fluostatins produced by the heterologous expression of a TAR reassembled environmental DNA derived type II PKS gene cluster. J. Am. Chem. Soc. 132, 11902–11903 (2010). 20690632 10.1021/ja104550p PMC2930618

[b4] Janso J. E. *et al.* Discovery of the lomaiviticin biosynthetic gene cluster in Salinispora pacifica. Tetrahedron 70, 4156–4164 (2014). 25045187 10.1016/j.tet.2014.03.009 PMC4101813

[b5] Kersten R. D. *et al.* Bioactivity-guided genome mining reveals the lomaiviticin biosynthetic gene cluster in Salinispora tropica. ChemBioChem 14, 955–962 (2013). 23649992 10.1002/cbic.201300147 PMC3755882

[b6] Colis L. C. *et al.* The cytotoxicity of (−)-lomaiviticin A arises from induction of double-strand breaks in DNA. Nat. Chem. 6, 504–510 (2014). 24848236 10.1038/nchem.1944 PMC4090708

[b7] Herzon S. B. & Woo C. M. The diazofluorene antitumor antibiotics: structural elucidation, biosynthetic, synthetic, and chemical biological studies. Nat. Prod. Rep. 29, 87–118 (2012). 22037715 10.1039/c1np00052g

[b8] Khdour O. & Skibo E. B. Quinone methide chemistry of prekinamycins: 13C-labeling, spectral global fitting and in vitro studies. Org. Biomol. Chem. 7, 2140–2154 (2009). 19421453 10.1039/b903844b

[b9] Feldman K. S. & Eastman K. J. Studies on the mechanism of action of prekinamycin, a member of the diazoparaquinone family of natural products: evidence for both sp2 radical and orthoquinonemethide intermediates. J. Am. Chem. Soc. 128, 12562–12573 (2006). 16984207 10.1021/ja0642616 PMC2515591

[b10] Laufer R. S. & Dmitrienko G. I. Diazo group electrophilicity in kinamycins and lomaiviticin A: potential insights into the molecular mechanism of antibacterial and antitumor activity. J. Am. Chem. Soc. 124, 1854–1855 (2002). 11866589 10.1021/ja0167809

[b11] Woo C. M., Ranjan N., Arya D. P. & Herzon S. B. Analysis of diazofluorene DNA binding and damaging activity: DNA cleavage by a synthetic monomeric diazofluorene. Angew. Chem. Int. Ed. 53, 9325–9328 (2014). 10.1002/anie.201404137 PMC4206835 25044348

[b12] Wang B. *et al.* Kinamycin biosynthesis employs a conserved pair of oxidases for B-ring contraction. Chem. Commun. (Camb.) 51, 8845–8848 (2015). 25920893 10.1039/c5cc01986a

[b13] Bunet R. *et al.* Characterization and manipulation of the pathway-specific late regulator AlpW reveals Streptomyces ambofaciens as a new producer of kinamycins. J. Bacteriol. 193, 1142–1153 (2011). 21193612 10.1128/JB.01269-10 PMC3067597

[b14] Pang X. *et al.* Functional angucycline-like antibiotic gene cluster in the terminal inverted repeats of the Streptomyces ambofaciens linear chromosome. Antimicrob. Agents Chemother. 48, 575–588 (2004). 14742212 10.1128/AAC.48.2.575-588.2004 PMC321545

[b15] Shen B. & Gould S. J. Opposite facial specificity for two hydroquinone epoxidases:(3-si, 4-re)-2, 5-dihydroxyacetanilide epoxidase from Streptomyces LL-C10037 and (3-re, 4-si)-2, 5-dihydroxyacetanilide epoxidase from Streptomyces MPP 3051. Biochemistry 30, 8936–8944 (1991). 1892811 10.1021/bi00101a004

[b16] Gould S. J. & Shen B. Epoxyquinones from 2, 5-dihydroxyacetanilide: opposite facial specificity in the epoxidation by enzymes from Streptomyces LL-C10037 and Streptomyces MPP 3051. J. Am. Chem. Soc. 113, 684–686 (1991). 10.1021/bi00101a004 1892811

[b17] Fahey R. C. Glutathione analogs in prokaryotes. Biochim. Biophys. Acta 1830, 3182–3198 (2013). 23075826 10.1016/j.bbagen.2012.10.006

[b18] Martinez Cuesta S., Furnham N., Rahman S. A., Sillitoe I. & Thornton J. M. The evolution of enzyme function in the isomerases. Curr. Opin. Struct. Biol. 26, 121–130 (2014). 25000289 10.1016/j.sbi.2014.06.002 PMC4139412

[b19] Präg A. *et al.* Regio- and stereoselective intermolecular oxidative phenol coupling in Streptomyces. J. Am. Chem. Soc. 136, 6195–6198 (2014). 24746278 10.1021/ja501630w

[b20] Ma J. *et al.* Biosynthesis of himastatin: assembly line and characterization of three cytochrome P450 enzymes involved in the post-tailoring oxidative steps. Angew. Chem. Int. Ed. 50, 7797–7802 (2011). 10.1002/anie.201102305 21726028

[b21] Young J. J., Ho S. N., Ju W. M. & Chang L. R. FL-120A-D', new products related to kinamycin from Streptomyces chattanoogensis subsp. taitungensis subsp. nov. II. Isolation and structure determination. J. Antibiot. 47, 681–687 (1994). 8040073 10.7164/antibiotics.47.681

[b22] Seaton P. J. & Gould S. J. New products related to kinamycin from Streptomyces murayamaensis. II. Structures of pre-kinamycin, keto-anhydrokinamycin, and kinamycins E and F. J. Antibiot. 42, 189–197 (1989). 2925510 10.7164/antibiotics.42.189

[b23] Kieser T., Bibb M. J., Buttner M. J., Chater K. F. & Hopwood D. A. Practical Streptomyces Genetics John Innes Foundation Norwich (2000).

[b24] Bradford M. M. A rapid and sensitive method for the quantitation of microgram quantities of protein utilizing the principle of protein-dye binding. Anal. Biochem. 72, 248–254 (1976). 942051 10.1016/0003-2697(76)90527-3

