# Supplementary material for: Identification of Alp1U and Lom6 as epoxy hydrolases and implications for kinamycin and lomaiviticin biosynthesis
Source: Nat Commun. 2015 Jul 2;6:7674. doi: 10.1038/ncomms8674 (PMC4506494; doi:10.1038/ncomms8674)
Supplement: Supplementary Information — Supplementary Figures 1-20 and Supplementary Tables 1-4 [file ncomms8674-s1.pdf]

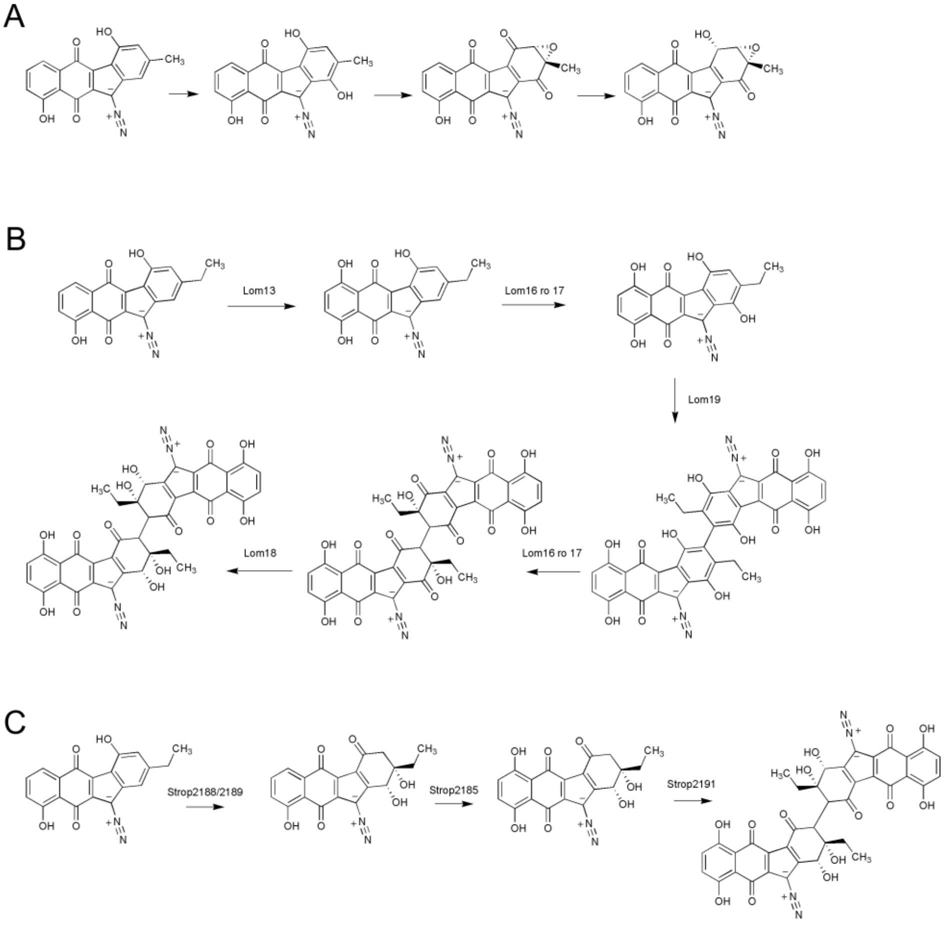

Supplementary Figure 1. The proposed biosynthetic pathways for the A-ring transformation of the kinamycin and lomaiviticin antibiotics. A, by Steven J. Gould; B, by Emily P. Balskus; C, by Bradley S. Moore.

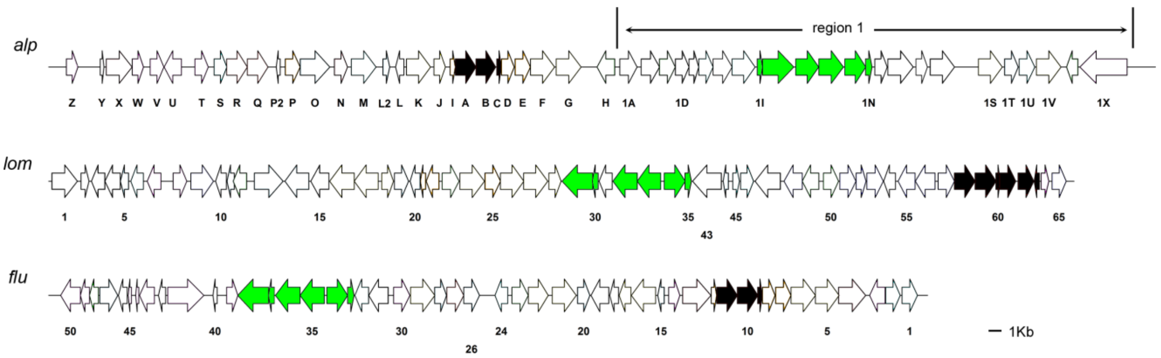

Supplementary Figure 2. Comparison of the *alp*, *lom*, and *flu* gene clusters. The three gene clusters are responsible for the biosynthesis of kinamycin, lomaiviticin and fluostatin, respectively. The DNA region labeled as "region 1" is extended in this study. The genes in black are indicated as mini-PKS, in green as the conserved six-gene subregion responsible for the diazo assembly.

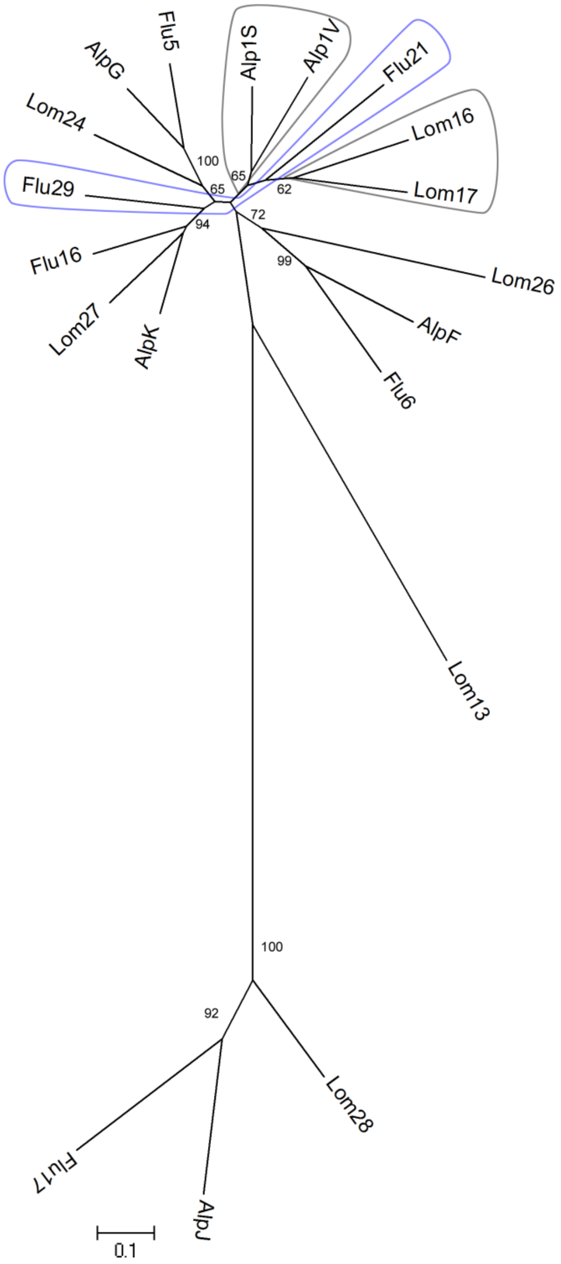

Supplementary Figure 3. Phylogenetic analysis of the oxygenases in the *alp*, *lom* and *flu* clusters. The tree was constructed by Neighbor-Joining method and the bootstrap (500 replicates) values for the main clades are shown. Scale bar represents 10% dissimilarity. The oxygenases labeled in black or blue are the candidates discussed in this study for A-ring transformation in the kinamycin, fluostatin, and lomaiviticin biosynthesis.

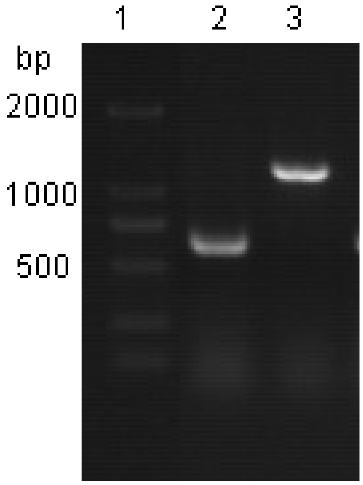

Supplementary Figure 4. PCR confirmation of *alp1U* double-deletion mutant. Lane 1, DNA ladder; lane 2, ΔΔ*alp1U* giving a band 631 bp. lane 3, the starting strain giving a band corresponding to a fragment of 1,204 bp in length. Both detected bands were correct in size.

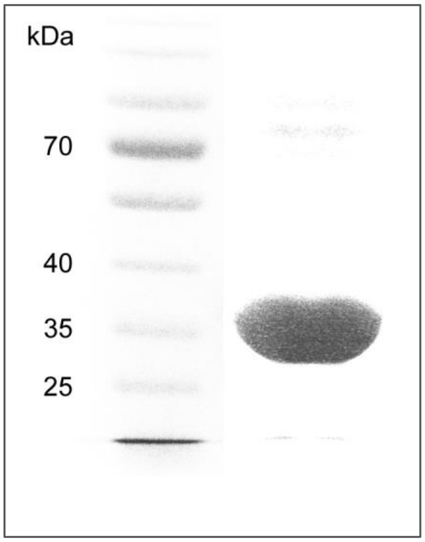

Supplementary Figure 5. SDS-PAGE analysis of the purified Alp1U. Lane 1, protein marker; lane 2, Alp1U giving a band corresponding to a protein of 37 kDa. The detected band was correct in size.

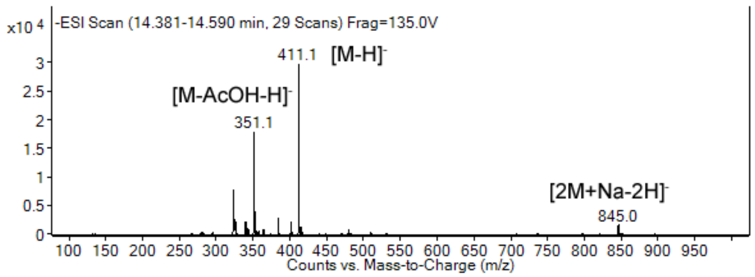

Supplementary Figure 6. LC-MS spectrum of kinamycin E (**5**) under ESI negative mode.

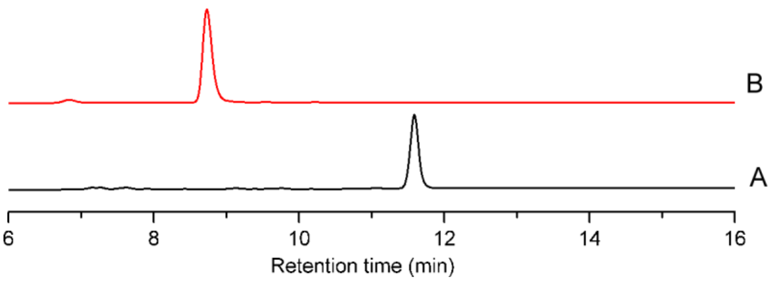

Supplementary Figure 7. HPLC analysis of kinamycin E (**5**) dissolved in $(CD_3)_2SO$. (A) before NMR measurement; (B) after NMR measurement.

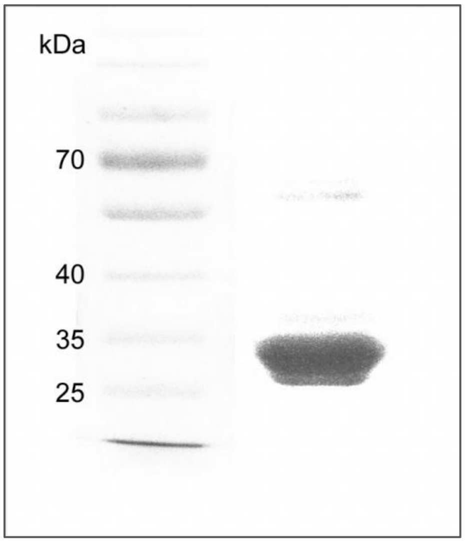

Supplementary Figure 8. SDS-PAGE analysis of the purified Lom6. Lane 1, protein marker; lane 2, Lom6 giving a band corresponding to a protein of 30 kDa. The detected band was correct in size.

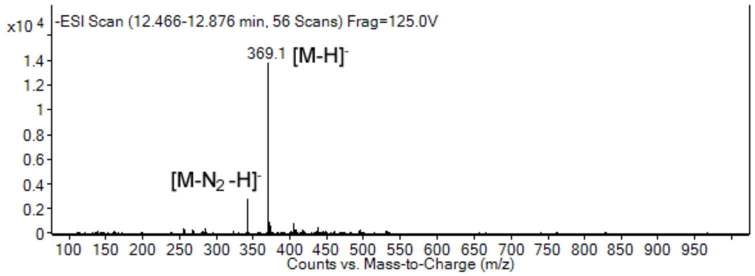

Supplementary Figure 9. LC-MS spectrum of kinamycin F (**2**) under ESI negative mode.

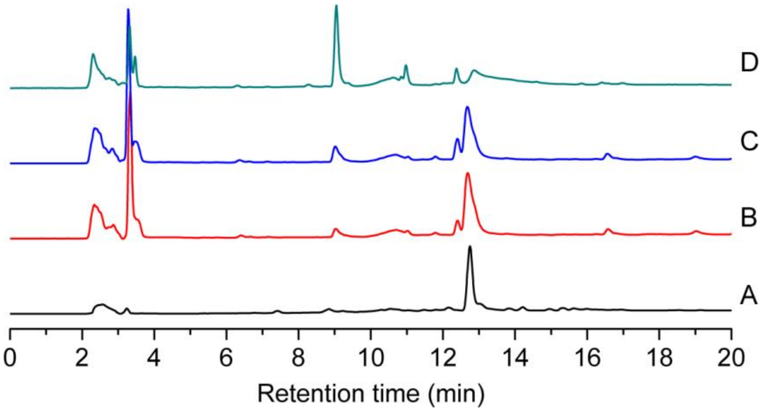

Supplementary Figure 10. HPLC profiles of Lom6 reaction with longer incubation time. (A) standard epoxykinamycin (**1**); (B) 10 min; (C) 20 min; (D) 50 min

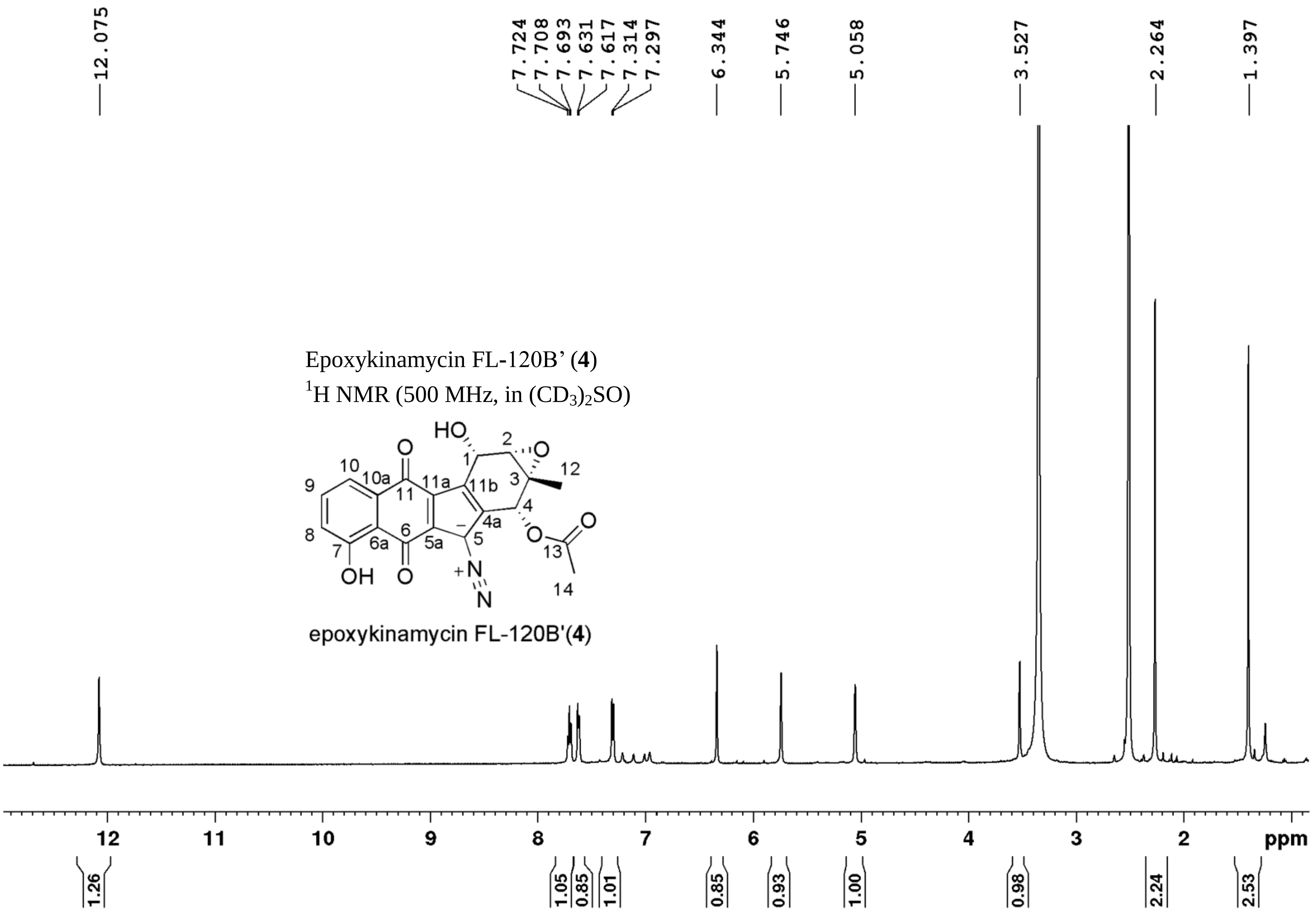

Supplementary Figure 11. ^{1}H NMR spectrum for epoxykinamycin FL-120B' (**4**).

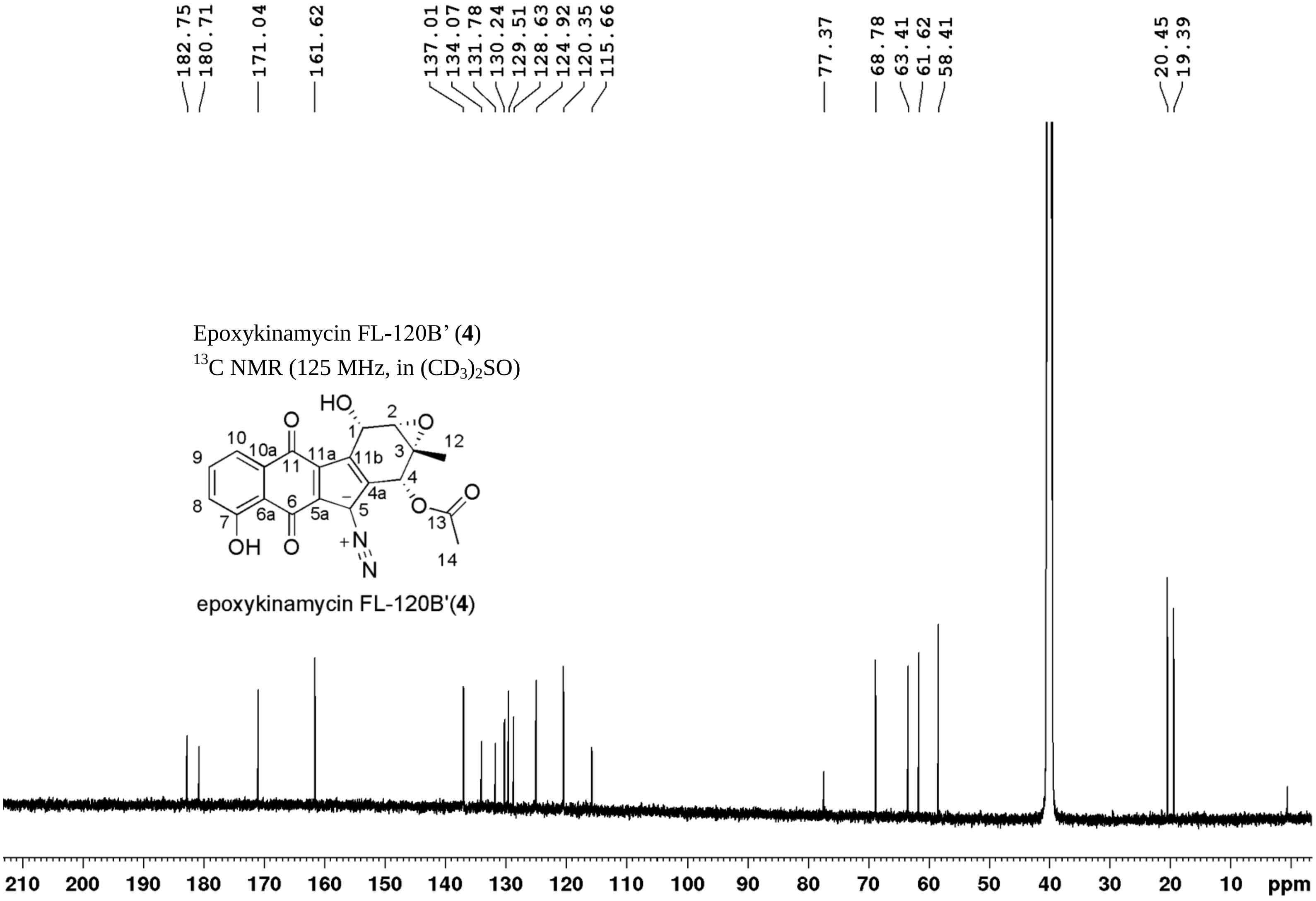

Supplementary Figure 12. ^{13}C NMR spectrum for epoxykinamycin FL-120B' (**4**).

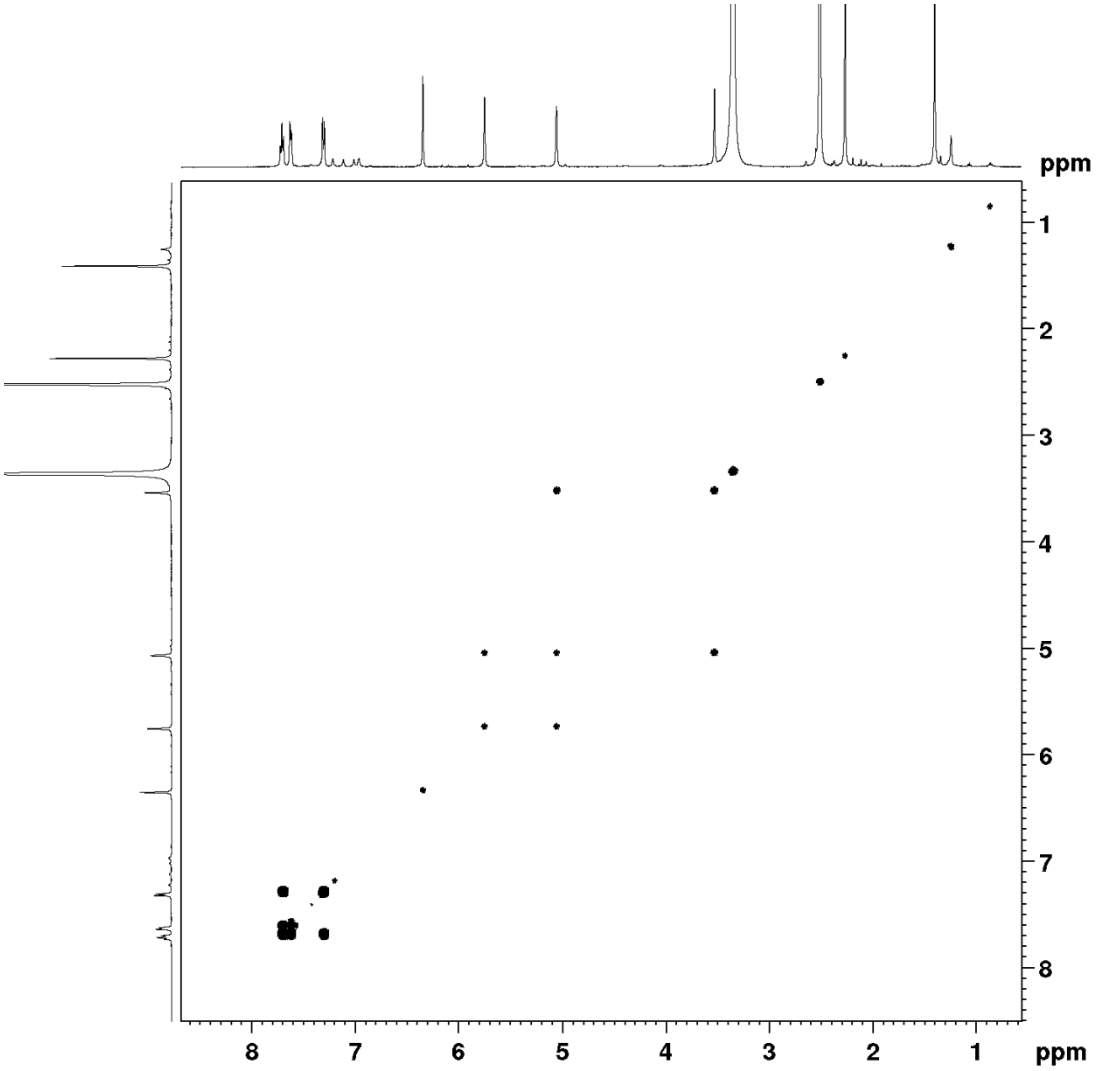

Epoxykinamycin FL-120B' (**4**)
COSY (500 MHz, in $(CD_3)_2SO$)

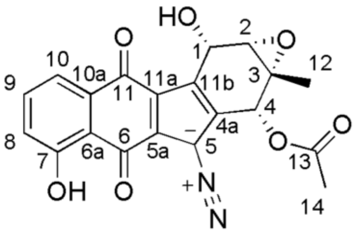

epoxykinamycin FL-120B'(**4**)

Supplementary Figure 13. COSY spectrum for epoxykinamycin FL-120B' (**4**).

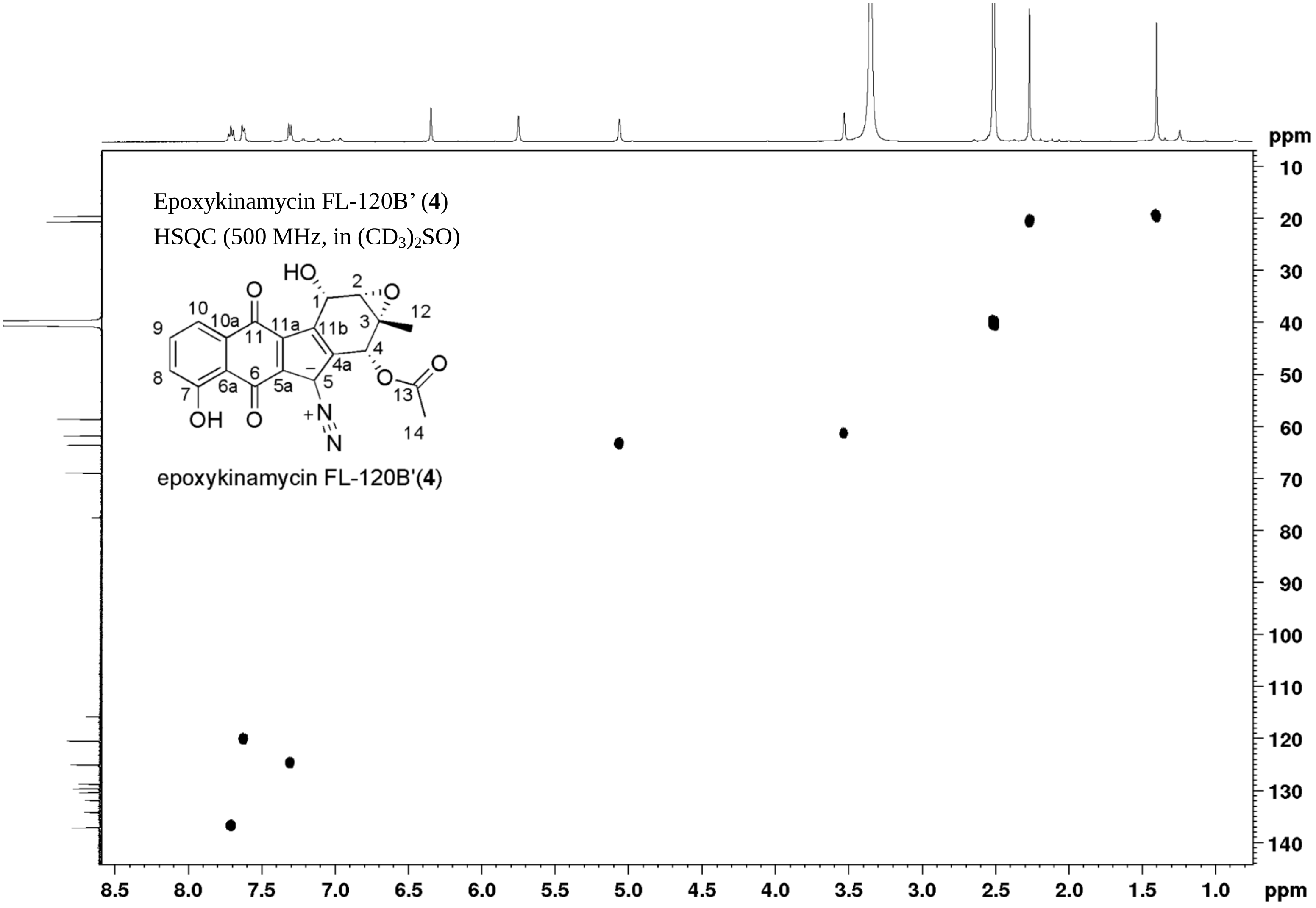

Supplementary Figure 14. HSQC spectrum for epoxykinamycin FL-120B' (**4**).

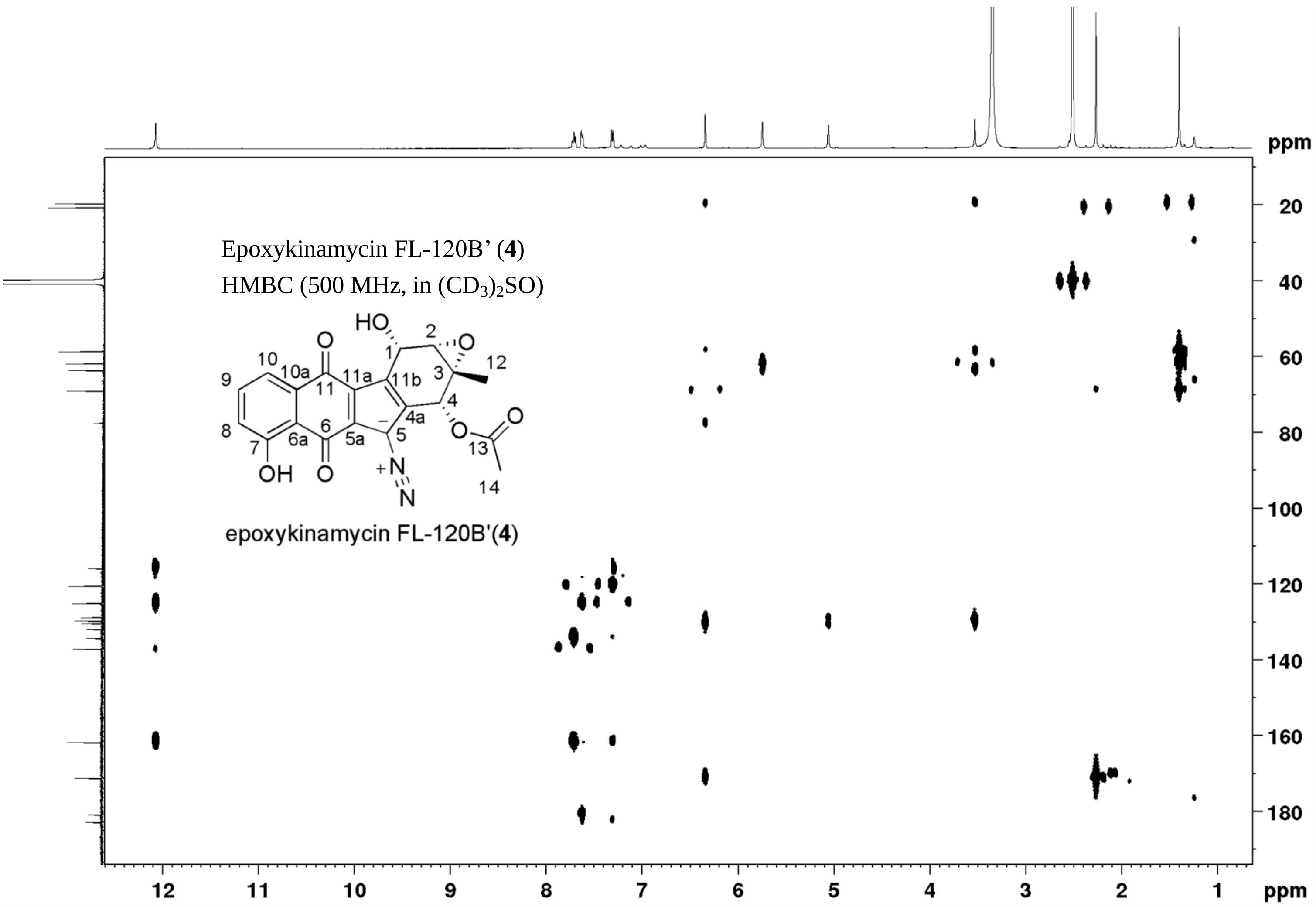

Supplementary Figure 15. HMBC spectrum for epoxykinamycin FL-120B' (**4**).

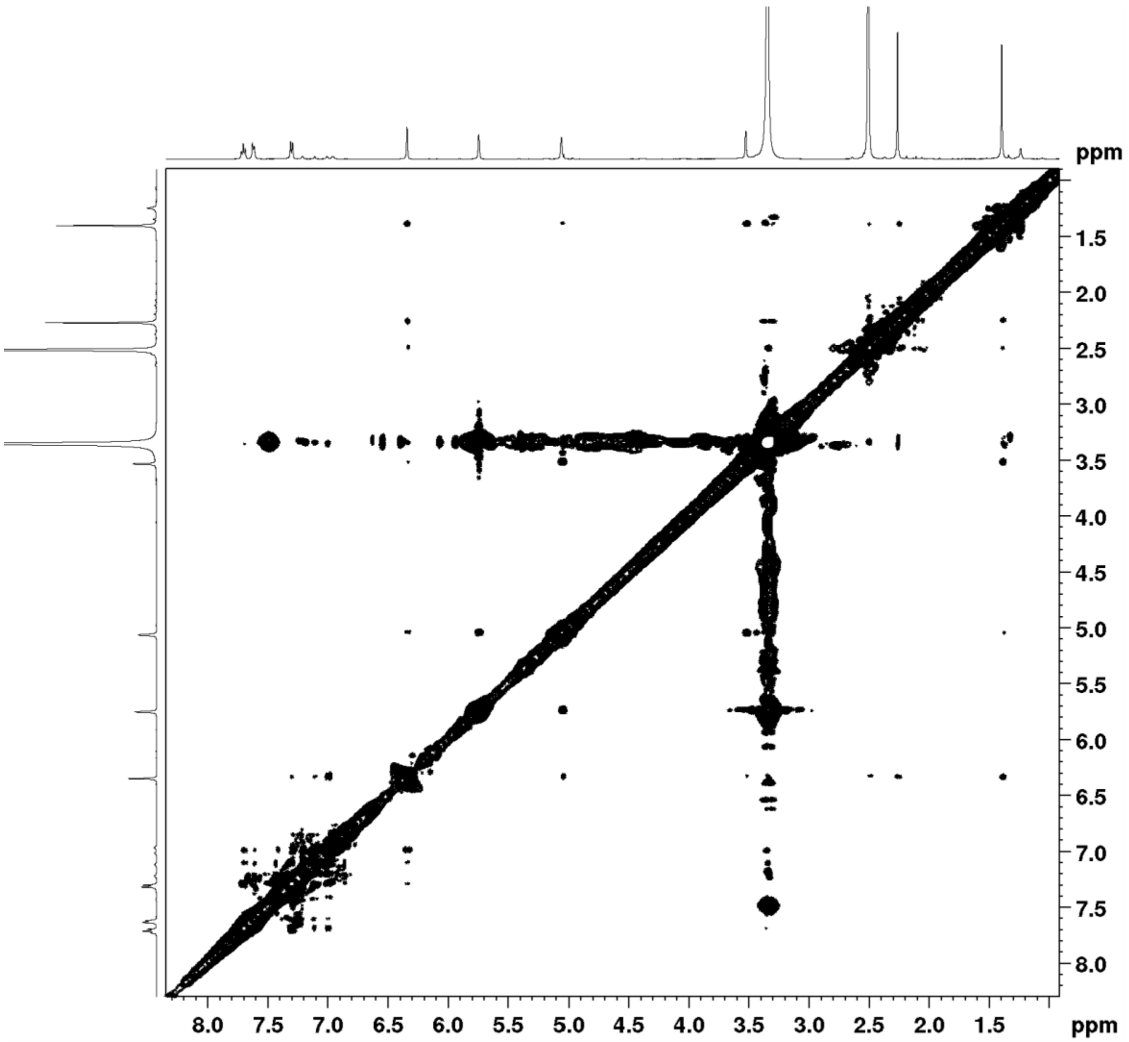

Epoxykinamycin FL-120B’ (**4**)

NOESY (500 MHz, in $(CD_3)_2SO$)

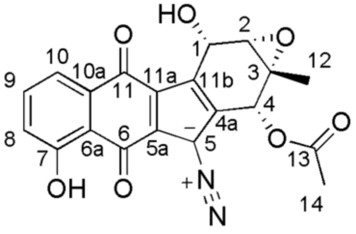

epoxykinamycin FL-120B'(**4**)

Supplementary Figure 16. NOESY spectrum for epoxykinamycin FL-120B’ (**4**).

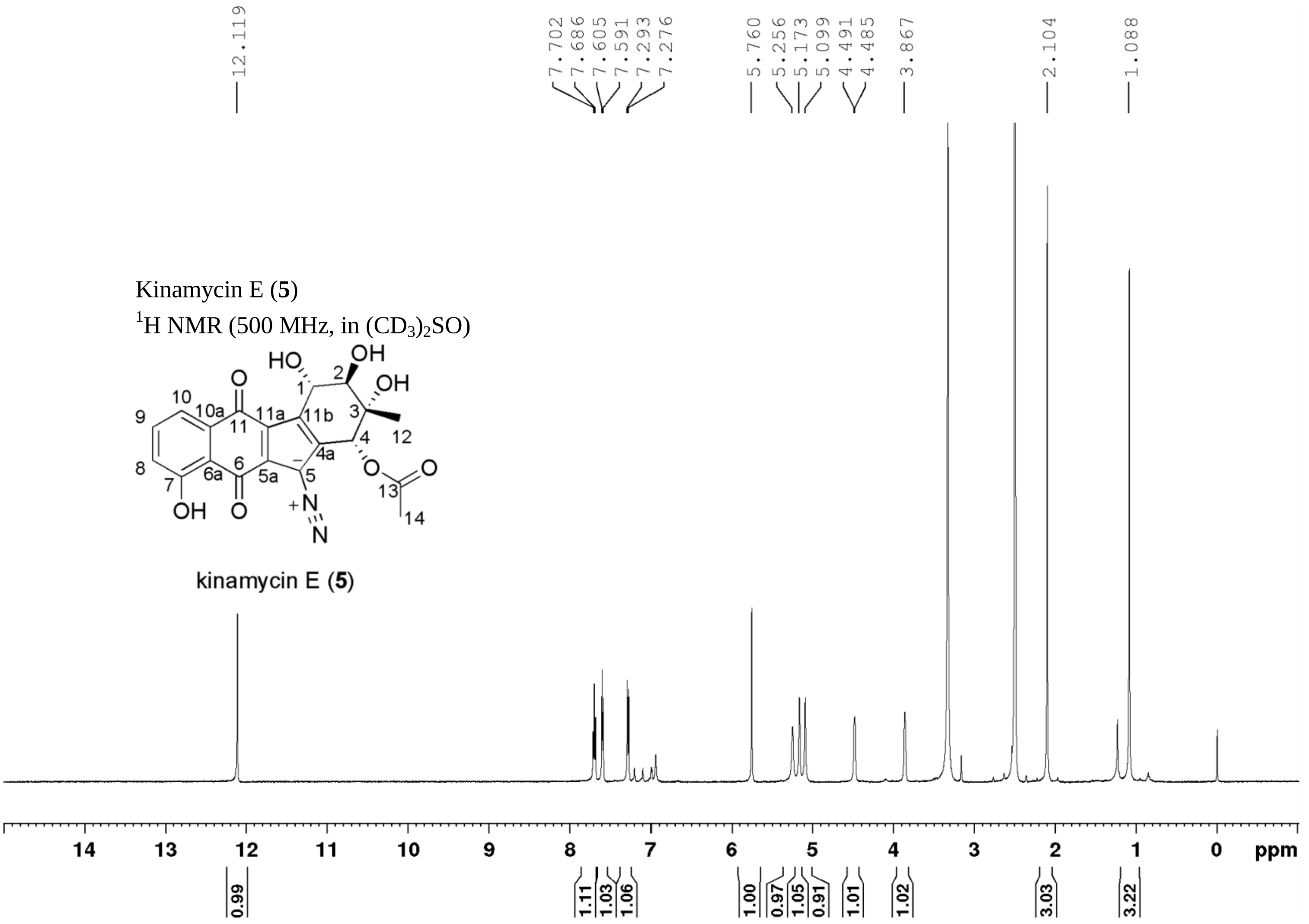

Supplementary Figure 17. ^{1}H NMR spectrum for kinamycin E (**5**).

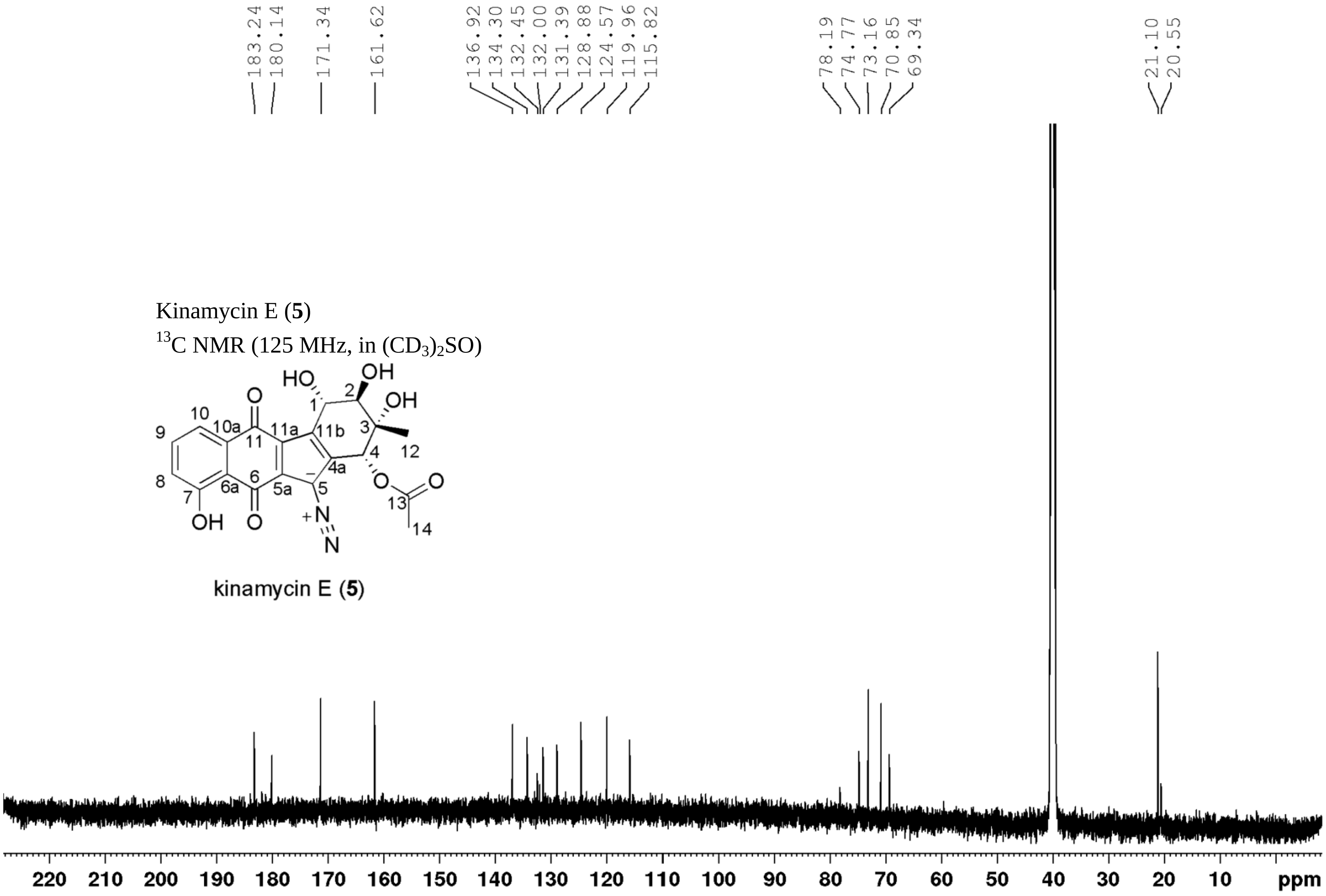

Supplementary Figure 18. ^{13}C NMR spectrum for kinamycin E (**5**).

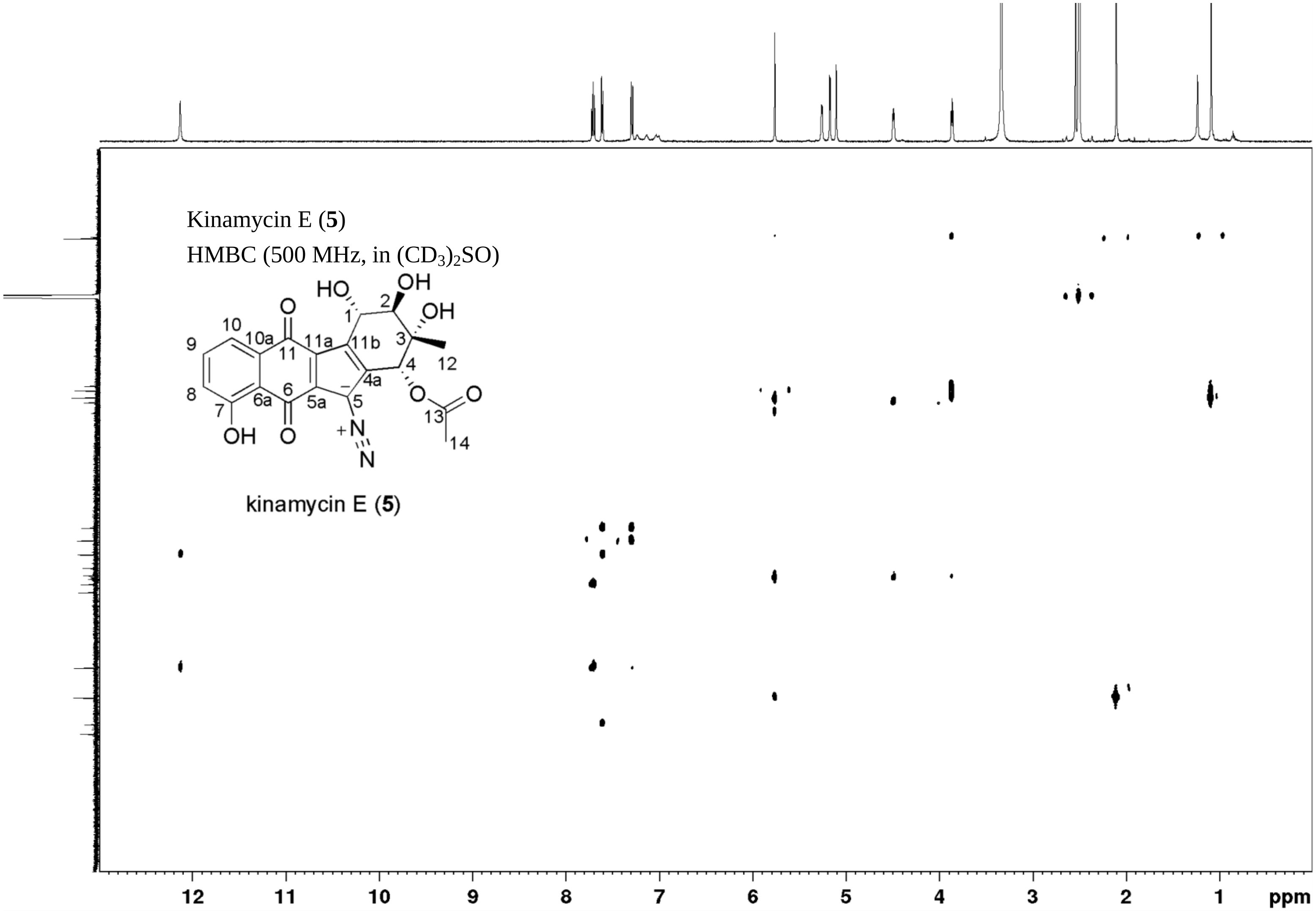

Supplementary Figure 19. HMBC spectrum for kinamycin E (**5**).

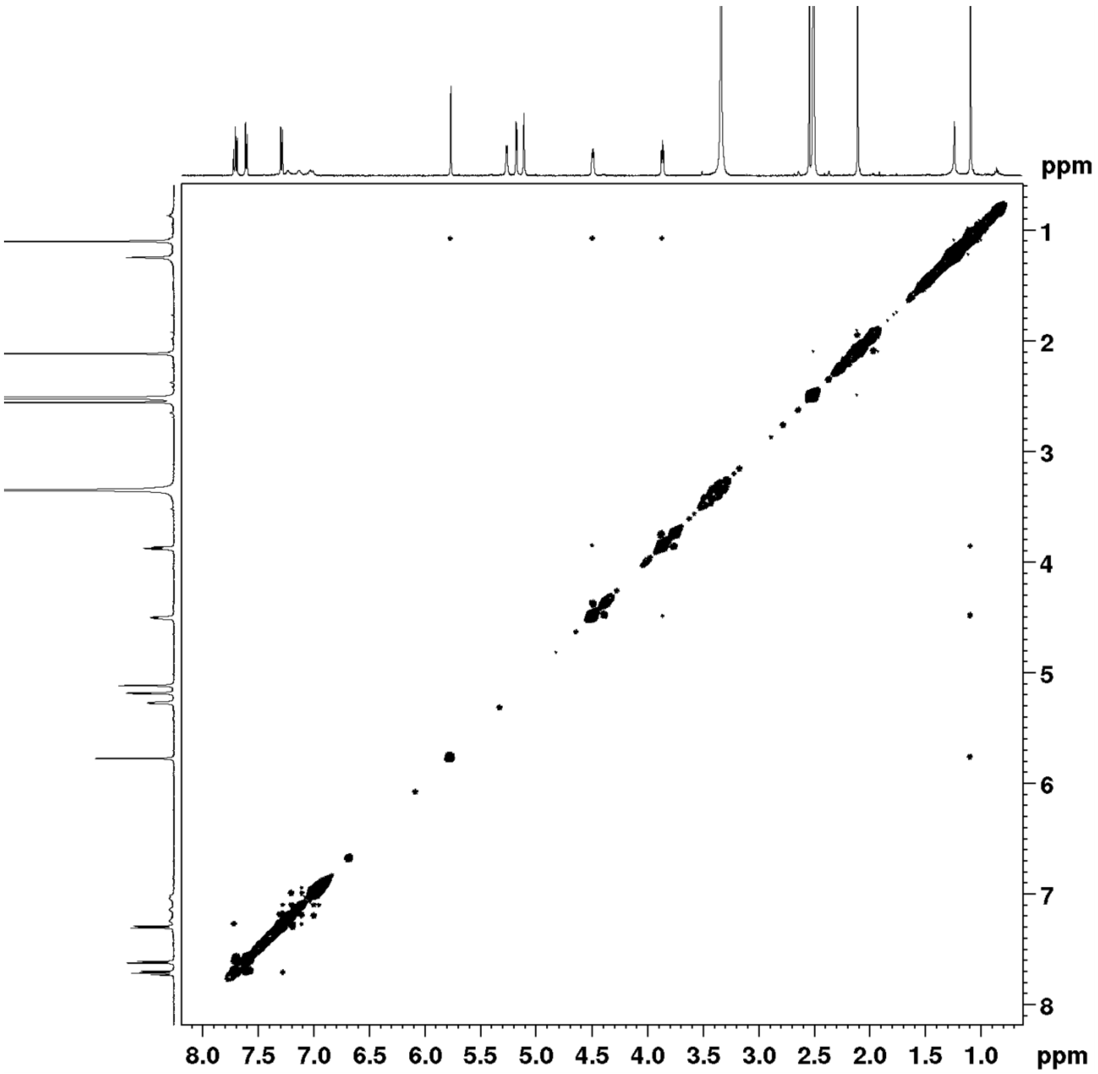

Kinamycin E (**5**)

NOESY (500 MHz, in $(CD_3)_2SO$)

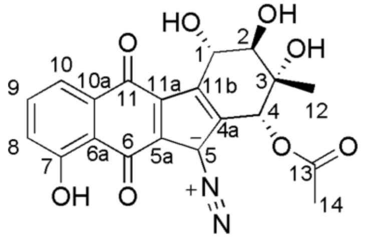

kinamycin E (**5**)

Supplementary Figure 20. NOESY spectrum for kinamycin E (**5**).

Supplementary Table 1. Predicted functions of the gene products in the *alp* cluster.

gene	predicted function	note
alpZ	gamma-butyrolactone binding protein	the previously proposed *alp* cluster
alpY	hypothetical protein	
alpX	putative carboxyl transferase	
alpW	TetR-family transcriptional regulator	
alpV	SARP family pathway specific regulator	
alpU	SARP family pathway specific regulator	
alpT	SARP family pathway specific regulator	
alpS	putative thioesterase	
alpR	putative polyketide synthase	
alpQ	putative chain length factor protein	
alpP2	hypothetical protein	
alpP	putative cyclase	
alpO	putative acyl-CoA dehydrogenase	
alpN	putative phosphopantetheinyl transferase	
alpM	peptide synthase condensation domain	
alpL2	conserved hypothetical protein	
alpL	JadX-like protein	
alpK	oxygenase	
alpJ	oxidase	
alpI	polyketide cyclase	
alpA	ketoacyl synthase	
alpB	chain length determinant	
alpC	acyl carrier protein	
alpD	polyketide ketoreductase	
alpE	cyclase/dehydratase	
alpF	oxygenase	
alpG	oxygenase	
alpH	putative O-methyltransferase	

Continued

gene	predicted function	note	
alp1A	conserved hypothetical protein	detoxification and DNA damage-repair	region 1
alp1B	putative myo-inositol phosphate synthase		
alp1C	putative transferase		
alp1D	putative abasic site repairing enzyme		
alp1E	conserved hypothetical protein		
alp1F	putative metal-dependent hydrolase		
alp1G	conserved hypothetical protein		
alp1H	putative type I phosphodiesterase		
alp1I	putative ferredoxin	diazo assembly machinery	
alp1J	hypothetical protein		
alp1K	putative amidase		
alp1L	putative glutamine synthetase		
alp1M	putative adenylosuccinate lyase		
alp1N	putative acetyltransferase		
alp1O	two-component system regulator	export and regulation	
alp1P	putative efflux protein		
alp1Q	conserved hypothetical protein		
alp1R	putative antibiotic antiporter		
alp1S	putative oxygenase	A-ring transformation	
alp1T	NDP-sugar epimerase		
alp1U	epoxy hydrolase		
alp1V	putative oxygenase		
alp1W	putative glutamine amidotransferase	diazo assembly machinery	
alp1X	putative transcriptional regulator		

Supplementary Table 2. The NMR data of epoxykinamycin FL-120B' (**4**)

Position	^{13}C-NMR[a]	^{1}H-NMR[b]	HMBC[b]	NOESY
1	63.4	5.06 (s, 1H)	C-4a	H-2, H-4, H_3-12
1-OH		5.69 (s, 1H)	C-1, C-2	
2	61.6	3.52 (t, 1H, *J* = 6.0 Hz)	C-1, C-3, C-11b, C-12	H_3-12
3	58.4			
4	68.8	6.34 (s, 1H)	C-3, C-5, C-11b, C-12	H_3-12
4a	129.5			
5	77.4			
5a	128.6			
6	182.8			
6a	115.6			
7	161.6			
7-OH		12.07 (s, 1H)	C-6a, C-7, C-8	
8	124.9	7.31 (d, 1H, *J* = 7.5 Hz)	C-6a, C-7, C-10	
9	137.0	7.72 (t, 1H, *J* = 7.5 Hz)	C-7, C-10a	
10	120.3	7.63(d, 1H, *J* = 7.5 Hz)	C-6a, C-8	
10a	134.1			
11	180.7			
11a	131.8			
11b	130.2			
12	19.4	1.40 (s, 3H)	C-2, C-3, C-4	
13	171.0			
14	20.4	2.26 (s, 3H)	C-13	

[a] Recorded at 125 MHz in $(CD_3)_2SO$. [b] Recorded at 500 MHz in $(CD_3)_2SO$.

Supplementary Table 3. The NMR data of kinamycin E (**5**)

Position	^{13}C-NMR^{a}	^{1}H-NMR^{b}	HMBC^{b}	NOESY
1	69.3	4.50 (dd, 1H, *J* = 3.5, 6.0 Hz)	C-3, C-4a	H-2, H-12
1-OH		5.18 (d, 1H, *J* = 3.5 Hz)		
2	74.8	3.87 (t, 1H, *J* = 6.0 Hz)	C-1, C-3, C-11b, C-12	H-1
2-OH		5.26 (d, 1H, *J* = 6.0 Hz)		
3	73.2			
3-OH		5.10 (s, 1H)		
4	70.8	5.76 (s, 1H)	C-2, C-5, C-11b, C-12	H-12
4a	132.4			
5	78.2			
5a	128.9			
6	183.2			
6a	115.8			
7	161.6			
7-OH		12.11 (s, 1H)	C-6a, C-7, C-8	
8	124.6	7.29 (d, 1H, *J* = 8.5 Hz)	C-6a, C-7, C-10	
9	136.9	7.70 (t, 1H, *J* = 8.5 Hz)	C-7, C-10a	
10	119.9	7.61(d, 1H, *J* = 8.5 Hz)	C-6a, C-8	
10a	134.3			
11	180.2			
11a	131.4			
11b	132.0			
12	20.6	1.09 (s, 3H)	C-2, C-3, C-4	
13	171.3			
14	21.1	2.10 (s, 3H)	C-13	

^{a} Recorded at 125 MHz in $(CD_3)_2SO$. ^{b} Recorded at 500 MHz in $(CD_3)_2SO$.

Supplementary Table 4. List of primers used in this study.

Primers	Restriction sites	Sequences
1U-upF	*Hin*dIII	5' CCCAAGCTTGTGCAGATCCCCGACGCC 3'
1U-upR	*Xba*I	5' GCTCTAGAGGCCAGTTCCCGGTCCGA 3'
1U-dnF	*Xba*I	5' GCTCTAGATACGCCAACGCCTACAACAGC 3'
1U-dnR	*Eco*RI	5' GGAATTCCCCATGTCGTAGCGGATGTC 3'
CK-1U-F		5' CGAGGACATGGGTAGATGACGC 3'
CK-1U-R		5' GGCCGGGCAGTTTGTCGTAGA 3'
1U-F	*Nde*I	5' GGAATTCCATATGCATGGGTAGATGACGCTGAAC 3'
1U-R	*Eco*RI	5' GGAATTCGAGGTGGCGTCAGCCGA 3'
Ealp1U-F	*Nde*I	5' GGAATTCCATATGCTCGCCCTGGGCACGG 3'
Ealp1U-R	*Hin*dIII	5' CCCAAGCTTGGAGGTGGCGTCAGCCGAAGAAG 3'
